# Staring Spotlight SAR with Nonlinear Frequency Modulation Signal and Azimuth Non-Uniform Sampling for Low Sidelobe Imaging

**DOI:** 10.3390/s21196487

**Published:** 2021-09-28

**Authors:** Wei Xu, Lu Zhang, Chonghua Fang, Pingping Huang, Weixian Tan, Yaolong Qi

**Affiliations:** 1 College of Information Engineering, Inner Mongolia University of Technology, Hohhot 010080, China; xuwei1983@imut.edu.cn (W.X.); 20191800049@imut.edu.cn (L.Z.); hwangpp@imut.edu.cn (P.H.); wxtan@imut.edu.cn (W.T.); qiyaolong@imut.edu.cn (Y.Q.); 2 Inner Mongolia Key Laboratory of Radar Technology and Application, Hohhot 010051, China; 3 Science and Technology on Electromagnetic Compatibility Laboratory, Wuhan 430064, China

**Keywords:** staring spotlight mode, nonlinear frequency modulation (NLFM), non-uniform sampling, low sidelobe imaging

## Abstract

In synthetic aperture radar (SAR) imaging, geometric resolution, sidelobe level (SLL) and signal-to-noise ratio (SNR) are the most important parameters for measuring the SAR image quality. The staring spotlight mode continuously transmits signals to a fixed area by steering the azimuth beam to acquire azimuth high geometric resolution, and its two-dimensional (2D) impulse response with the low SLL is usually obtained from the 2D weighted power spectral density (PSD) by the selected weighting window function. However, this results in the SNR reduction due to 2D amplitude window weighting. In this paper, the staring spotlight SAR with nonlinear frequency modulation (NLFM) signal and azimuth non-uniform sampling (ANUS) is proposed to obtain high geometric resolution SAR images with the low SLL and almost without any SNR reduction. The NLFM signal obtains non-equal interval frequency sampling points under uniform time sampling by adjusting the instantaneous chirp rate. Its corresponding PSD is similar to the weighting window function, and its pulse compression result without amplitude window weighting has low sidelobes. To obtain a similar Doppler frequency distribution for low sidelobe imaging in azimuth, the received SAR echoes are designed to be non-uniformly sampled in azimuth, in which the sampling sequence is dense in middle and sparse in both ends, and azimuth compression result with window weighting would also have low sidelobes. According to the echo model of the proposed imaging mode, both the back projection algorithm (BPA) and range migration algorithm (RMA) are modified and presented to handle the raw data of the proposed imaging mode. Both imaging results on simulated targets and experimental real SAR data processing results of a ground-based radar validate the proposed low sidelobe imaging mode.

## 1. Introduction

High geometric resolution, low sidelobe level (SLL), and high signal-to-noise ratio (SNR) have always been pursued in synthetic aperture radar (SAR) data acquisition and imaging processes [1,2,3]. The high resolution range is determined by a transmitted signal with the large pulse bandwidth, while the high resolution azimuth is implemented by increasing the synthetic aperture time. The spotlight SAR, including sliding spotlight and starring spotlight modes, can achieve high-resolution imaging via azimuth beam steering from fore to aft during the whole SAR acquisition interval, and the azimuth resolution is determined by the azimuth beam steering capacity [4,5,6]. For a certain azimuth beam steering angle, the staring spotlight mode can provide the highest azimuth resolution, which sets the azimuth beam rotation center in the imaged scene center and always illuminates a fixed imaged area. The starring spotlight mode has been practically applied in the TerraSAR-X satellite, which can improve the azimuth resolution to about 0.2 m [7,8]. In addition to the high geometric resolution, low SLL and high SNR are two other important performances to measure the SAR image quality. However, there has always been a contradiction between the low SLL and the high SNR, since the low sidelobes are usually obtained by window weighting during focusing process, which results in the SNR reduction in the focused SAR images [9,10,11].

To avoid the SNR reduction caused by window weighting in the pulse compression, the nonlinear frequency modulation (NLFM) waveform instead of the linear frequency modulation (LFM) waveform is suggested to be transmitted [2,12], as its discrete frequency samples are non-uniformly distributed and its corresponding power spectral density (PSD) is sharp weighted. The use of the NLFM signal is equivalent to the transfer of the window function required for weighting of the LFM signal to the transmitting system, and there is no need to use a weighting network during range pulse compression. In the NLFM signal generation, the group delay of the NLFM waveform is mainly calculated according to the principle of stationary phase (POSP) [13,14], and the range frequency non-uniform distribution could be obtained from the inverse function of the group time delay. In other words, the range time is uniformly sampled, and the time-frequency function of the generated NLFM signal changes the instantaneous frequency modulation rate of the NLFM signal.

In SAR imaging, the azimuth signal could be approximately regarded as the chirp signal. Similar to the NLFM signal, the non-uniform frequency distribution should be obtained for sidelobe suppression without window weighting. The method to obtain the non-uniform frequency distribution via adjusting the frequency modulation rate cannot be implemented in azimuth, since the Doppler modulation rate is only related to the imaging geometry and cannot be easily changed [15,16]. The way to obtain the non-uniform Doppler frequency distribution is by using azimuth non-uniform sampling (ANUS) [17,18,19]. For all imaged targets, the sampling points at the center of the synthetic aperture time are denser, and the sampling points in both sides are sparse. This sampling behavior means that the Doppler frequency will change slowly in the middle and fast in both sides, which is similar to the NLFM generation. To obtain such a Doppler frequency sampling distribution for all targets in the imaged scene, each target should have the same start and end time of the synthetic aperture, and the starring spotlight mode meets with this requirement. Therefore, the novel starring spotlight mode with NLFM signal and ANUS is proposed for low sidelobe imaging. The raw data of the proposed imaging mode could be focused with low sidelobes without any amplitude weighting, and the SNR loss due to sidelobe suppressions is avoided. However, it should be noted that azimuth Fourier transform (FT) can’t be directly applied due to azimuth non-uniform sampling. Therefore, the existing range migration algorithm (RMA) [20] should be adjusted to process the raw data of the proposed mode. Then, echoes can be well focused after range and azimuth preprocessing.

This paper is structured as follows. In Section 2, the method of generating the NLFM signal based on the POSP is briefly reviewed, and ANUS for the low SLL is proposed with based on the similar principle. In Section 3, the starring spotlight mode with NLFM and ANUS is proposed for low sidelobe imaging without window weighting, and both back projection algorithm (BPA) and range migration algorithm (RMA) are modified to process echoes of the proposed imaging mode. In Section 4, simulation experiments on both point and distributed targets are carried out, and a ground-based radar experiment is designed to validate the proposed low sidelobe imaging mode. Finally, conclusions are drawn in Section 5.

## 2. NLFM Signal and Azimuth Non-Uniform Sampling

**Figure 1:**
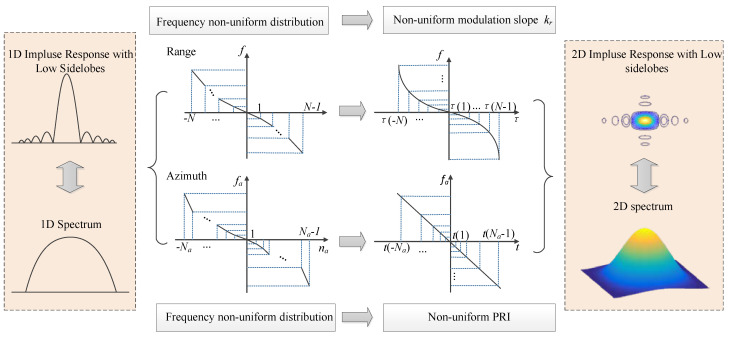
Sidelobe suppression without window weighting in range and azimuth dimensions.

To obtain the impulse response with low sidelobes, amplitude weighting by a selected window function during pulse compression is usually applied, which will make the spectrum shape of the LFM signal similar with taper weighting as shown in on the left side of Figure 1. Since window weighting changes the spectrum shape of the LFM signal and noise, according to the principle of the matched filter, the output SNR after pulse compression is not the maximum value, and window weighting results in the SNR loss in focused SAR images [3,21]. An improved method to obtain the tapering shape spectrum for the impulse response with low sidelobes is to generate the non-uniform discrete frequency distribution as shown in the middle part of Figure 1.

### 2.1. Review of NLFM Design

To obtain the one-dimension (1D) impulse response with low sidelobes without window weighting in the range dimension, the NLFM signal is suggested to be transmitted. The instantaneous frequency of the NLFM waveform is a nonlinear function of the range time. If the NLFM waveform is uniformly sampled in the range time domain, its corresponding range frequency samples are non-uniformly distributed. As shown in Figure 1, **f** represents the non-uniform discrete range frequency vector as follows:

(1) $$f=\left[f(-N),f(-N+1),\ldots ,f(N-1)\right]$$

where the number of sampling points of the transmitted NLFM pulse is 2*N*.

At present, the most commonly used method is to use the POSP to design the NLFM waveform. To obtain the impulse with low sidelobes, the PSD of the designed NLFM waveform should be with the same shape of the predefined window function such as Cosine, Kaiser and other tapering window functions. In the NLFM waveform design, the instantaneous frequency *f*(*τ*) is the inverse function of the group time delay function *τ*(*f*), and *τ*(*f*) can be expressed as follows:

(2) $$\tau (f)=Q_{r}\int_{-\infty }^{f}W(x)\cdot dx$$

with

(3) $$Q_{r}=\frac{T_{p}}{\int_{-B_{r}/2}^{B_{r}/2}W\left(f\right)df}$$

where *W*( ) is the selected weighting function, *T_p_* is the transmitted pulse duration, and *B_r_* is the pulse bandwidth.

With the time delay group function *τ*(*f*), the non-uniform discrete range frequency vector **f** could be easily obtained by interpolation processing. Furthermore, the continuous range frequency *f*(*τ*) in NLFM could be expanded in a series of polynomials for the NLFM generation and expressed as:

(4) $$f(\tau )=p_{0}+p_{1}\tau +p_{2}\tau^{2}+p_{3}\tau^{3}+\cdots +p_{M}\tau^{M}$$

where $p_{m}(m=0,1,2,\ldots ,M)$ denotes the polynomial coefficient, and *M* is the polynomial order. In the NLFM waveform, the waveform is usually uniformly sampled, and the instantaneous frequency modulation slope $k_{r}(\tau )$ is written as follows:

(5) $$k_{r}(\tau )=\frac{\partial f(\tau )}{\partial \tau }=p_{1}+p_{2}\tau +p_{3}\tau^{2}+\cdots +p_{M}\tau^{M-1}$$

Therefore, the non-uniform discrete range frequency vector **f** in the NLFM waveform is obtained by adjusting the instantaneous frequency modulation rate when the range time is uniformly sampled as shown in Figure 1.

**Figure 2:**
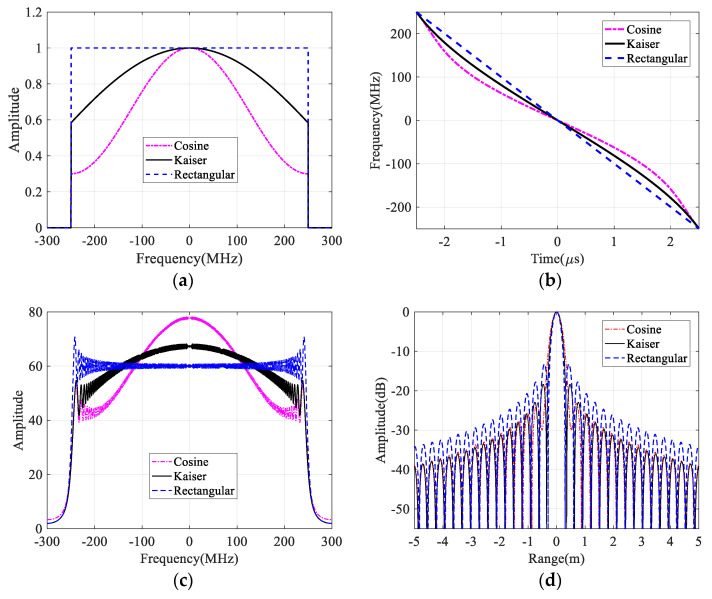
NLFM waveform design results with different types of window functions. (**a**) Different window functions. (**b**) Time–frequency relationships of designed waveforms. (**c**) Spectra of designed waveforms. (**d**) Pulse compression results.

In this paper, three window functions including rectangular, Kaiser, and cosine window functions are selected to design the NLFM waveform. The Kaiser window function is expressed as:

(6) $$w(n)=\frac{I_{0}\left[\beta \sqrt{1-{\left(1-\frac{2n}{N_{w}-1}\right)}^{2}}\right]}{I_{0}(\beta )},0\leq n\leq N_{w}$$

where $I_{0}(\cdot )$ is the first kind of zero-order Bessel function, and the window function factor *β* is used to adjust the shape of the Kaiser window. The raised cosine window function can also adjust the sidelobe level by adjusting the factor *α*, which can be expressed as:

(7) $$w(n)=\alpha +(1-\alpha )\cos\frac{\pi n}{N_{w}},\;0\leq n\leq N_{w}$$

where α∈[0,1] is the roll-off coefficient of the raised cosine window function. Figure 2 shows the NLFM waveform design results with different window functions. According to (2), different range time-frequency relationships are obtained from different window functions as shown in Figure 2b, while the rectangle function is related to the LFM waveform. When cosine and Kaiser window functions are introduced to generate the NLFM waveform, the resulting nonlinear range time-frequency relationship is obtained as shown in Figure 2b, and their corresponding spectra and pulse compression results are shown in Figure 2c,d respectively. Compared with the pulse compression result of the LFM waveform, the SLL is obviously reduced.

**Figure 3:**
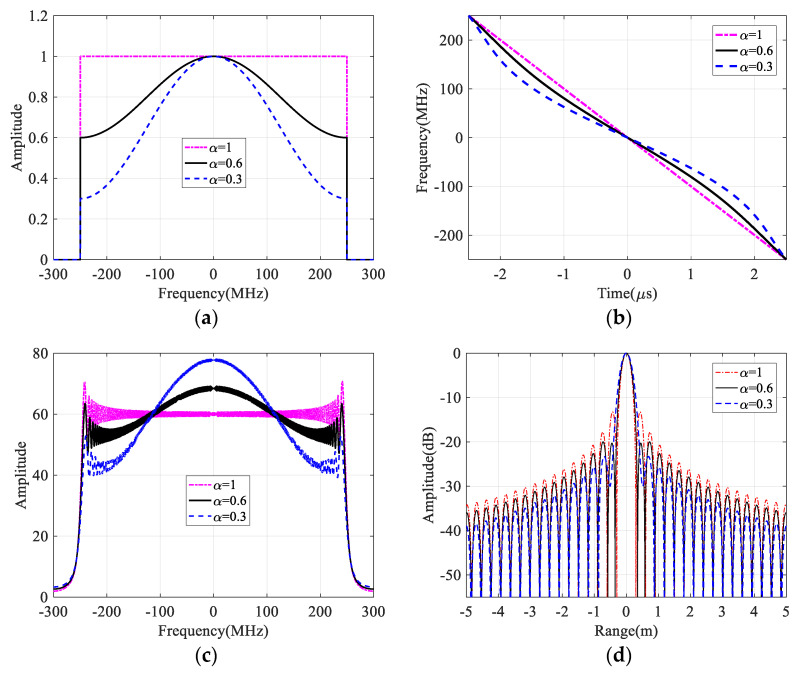
NLFM waveform design results with cosine window functions. (**a**) Raised cosine window functions with different roll–off coefficients. (**b**) Time–frequency relationships of designed waveforms. (**c**) Spectra of designed different waveforms. (**d**) Pulse compression results of designed different signals.

Since the cosine window function is widely adopted in practice for SAR imaging processes, Figure 3 shows NLFM waveform design results based on the cosine window function with different roll-off coefficients, while the roll-off coefficient *α* of the cosine window function is 1, 0.6, and 0.3, respectively. With the decrease of the roll-off coefficient, the time-frequency nonlinearity is more obvious, which results in the wanted low SLL as shown in Figure 3. Therefore, the non-uniform range frequency distribution could be obtained and adjusted by the chosen window function, and then the instantons frequency modulation rate is continuously changed during the whole NLFM generation interval as shown at the top of Figure 1, which brings a low SLL without amplitude weighting and avoid the SNR reduction in the final compressed result.

### 2.2. Azimuth Non-Uniform Sampling

In conventional SAR imaging, a radar platform with a small aperture antenna moves relative to the imaged target within the synthetic aperture time. The radar signal is periodically transmitted with a fixe pulse repetition frequency (PRF), and their corresponding echoes are received at different positions and coherently processed to obtain a higher azimuth resolution. Consequently, the azimuth signal can also be regarded as a chirp signal and sampled with PRF.

Similar to the NLFM in the range dimension, the desired non-uniform discrete Doppler frequency vector **f_a_** for low sidelobes in azimuth is expressed as follows:

(8) $$f_{a}=\left[f_{a}(-N_{a}),f_{a}(-N_{a}+1),\ldots ,f_{a}(n_{a}),\ldots ,f_{a}(N_{a}-1)\right]$$

where *n_a_* = −*N_a_*, −*N_a_* + 1, …, *N_a_* − 1 denotes the azimuth sample index, and the number of azimuth samples is 2*N_a_*. With the similar principle of the NLFM waveform generation, the instantaneous Doppler frequency *f_a_*(*t*) could be obtained as follows:

(9) $$f_{a}(t)=f^{-1}\left(t(f_{a})\right)$$

where *f*^−1^(·) indicates the inverse function operator. The azimuth sampling time can be expressed as:

(10) $$t(f_{a})=Q_{a}\int_{-\infty }^{f_{a}}W(x)\cdot dx$$

with

(11) $$Q_{a}=\frac{T_{s}}{\int_{-B_{a}/2+f_{ac}}^{B_{a}/2+f_{ac}}W\left(f_{a}\right)df_{a}}$$

where *f_ac_* is the Doppler center frequency of the target, similar to the continuous range frequency *f*(*τ*) in (4). The discrete Doppler frequency could be expanded in a series of polynomials as:

(12) $$f_{a}(n_{a})=q_{0}+q_{1}n_{a}+q_{2}n_{a}^{2}+q_{3}n_{a}^{3}+\cdots +q_{M}n_{a}^{M}$$

As the Doppler modulation rate is only related to the platform velocity and the slant range, it is very difficult to change the frequency modulation rate to obtain the desired non-uniform discrete Doppler frequency vector. Different from the generation of the non-uniform distributed range frequency vector **f**, the discrete Doppler frequency vector **f_a_** is obtained by azimuth non-uniform sampling, and the azimuth non-uniform sampled time is expressed as:

(13) $$t_{a}(n_{a})=\frac{f_{a}(n_{a})}{\left|k_{a}\right|}=\frac{q_{0}+q_{1}n_{a}+q_{2}n_{a}^{2}+q_{3}n_{a}^{3}+\cdots +q_{M}n_{a}^{M}}{\left|k_{a}\right|}$$

with

(14) $$k_{a}=-\frac{2v^{2}\cos^{3}\theta_{s}}{\lambda R_{0}}$$

where *k_a_* is the azimuth modulation frequency rate, *v* is the radar sensor velocity, *θ_s_* is the squint angle, *λ* is the wavelength, and *R*_0_ is the slant range from the targets to the sensor flight path. The non-uniform sampling time of the azimuth can be obtained according to the azimuth modulation frequency rate.

**Figure 4:**
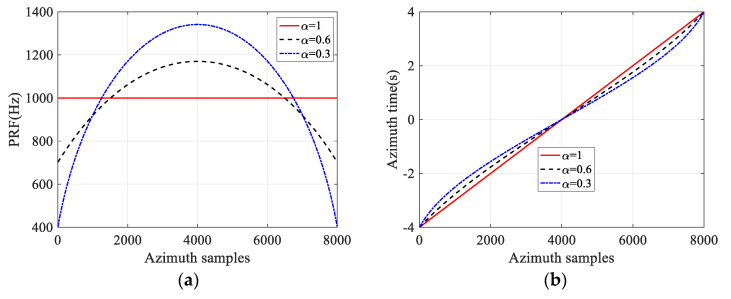

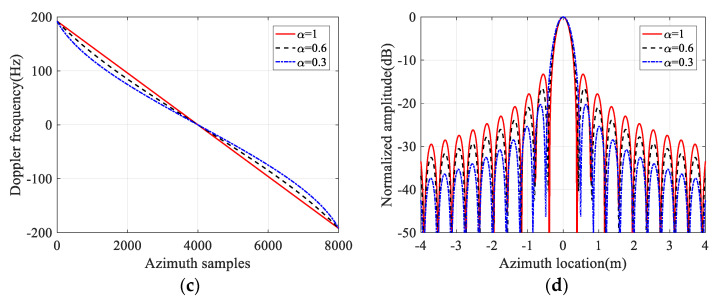
ANUS design results based on the selected raised cosine window function. (**a**) Continuous PRF Variation. (**b**) Azimuth time non-uniform sampling. (**c**) Doppler frequency sampling distribution. (**d**) Azimuth pulse compression results based on different ANUS design results.

Figure 4 shows the ANUS design results for sidelobe suppression based on the selected raised cosine window function. With the factor *α* = 0.6, the operated PRF is from 702.2 Hz to 1170.1 Hz, while the required PRF is 402.8~1341.0 Hz when the factor *α* is 0.3. Furthermore, the operated PRF is with a relative small value in both ends of the synthetic aperture interval and relative high in the middle as shown in Figure 4a. The high operated PRF corresponding to the small pulse repetition interval (PRI) means the dense sampling, while the low PRF indicates the sparse sampling as shown in Figure 4b. Consequently, the desired non-uniform discrete Doppler frequency distribution in Figure 1 is obtained as shown in Figure 4c, which results in suppressed sidelobes in the azimuth impulse response without amplitude weighting as shown in Figure 4d.

## 3. High Resolution Low Sidelobe Imaging

### 3.1. Starring Spotlight SAR with NLFM and ANUS

Sidelobes suppression is very important in SAR imaging, and amplitude weighting with a selected window function is usually adopted to obtain the low SLL. However, window weighting in the pulse compression leads to the SNR reduction due to mismatch in the matched filtering. The NLFM waveform is suggested to be transmitted and instead of the conventional LFM waveform, since the shape of its corresponding PSD is similar to the tapering window, which results in suppressed sidelobes without amplitude weighting. The major shortcoming of NLFM is sensitive to Doppler frequency shift, and it can be ignored for airborne SAR, since the Doppler bandwidth is very small compared to the pulse bandwidth.

To obtain the PSD with the window function shape in azimuth for all targets in the imaged scene, the operated PRF should be with a relative small value in both ends of the synthetic aperture interval and be relative high in the middle as shown in Figure 4a. This ANUS behavior cannot be implemented in the conventional stripmap/sliding spotlight mode. For these two modes, imaged targets at different azimuth positions have different synthetic aperture start and end times. In the starring spotlight mode, the radar illuminates the same spot on the ground, and all targets in the imaged scene have the same azimuth illumination history during the whole acquisition interval to obtain an ultra-high azimuth resolution. Consequently, the starring spotlight mode could implement the ANUS behavior for all targets at the same time, in which the sampling points at the center of the synthetic aperture time are denser and the sampling points on both sides are sparse as shown in Figure 4.

**Figure 5:**
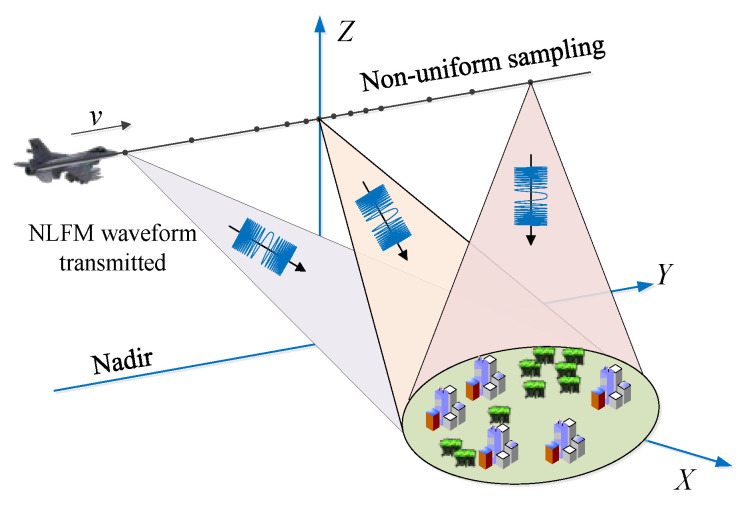
Imaging geometry of starring spotlight with NLFM waveform and ANUS.

Both NLFM and ANUS could overcome the limitation between the low SLL and the SNR reduction due to window weighting in focusing processes. Based on this phenomenon, the starring spotlight mode with NLFM and ANUS for low sidelobe imaging is proposed as shown in Figure 5. The radar continuously transmits the NLFM waveform to the imaged area with the non-uniform PRF, while the operated PRF is high in the middle and low in both ends of the SAR raw data acquisition interval. The 2D PSD shape of the prosed imaging mode is similar to the 2D weighting window function, which corresponds to the 2D impulse response with low sidelobes as shown in Figure 1.

### 3.2. SNR Loss Due to Window Weighting

**Figure 6:**
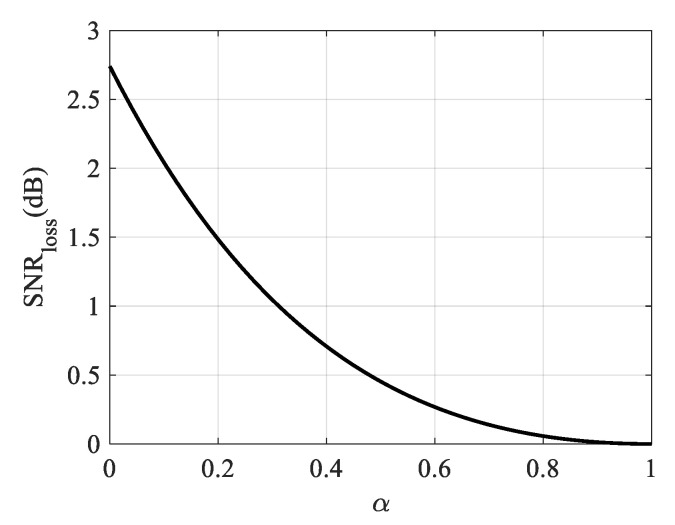
SNR loss caused by windowing during pulse compression.

In the range pulse compression, window weighting for sidelobe suppression usually leads to 1–2 dB SNR loss. The output SNR after conventional pulse compression can be expressed as [2,3]:

(15) $$\mathrm{SNR}=\frac{{\left|\int_{-\infty }^{+\infty }s(t_{0}-\tau )h(\tau )d\tau \right|}^{2}}{N_{0}\int_{-\infty }^{+\infty }{\left|h(\tau )\right|}^{2}d\tau }$$

where s(τ) represents the received echo, $t_{0}$ indecates the time delay for the detected target, h(τ) denotes the impulse response function of the matched filter for range compression, and *N*_0_ is the density of the power spectrum of noise. After the windowed pulse compression, the spectrum envelope is sharpened and the output SNR can be expressed as:

(16) $${\mathrm{SNR}}_{\omega }=\frac{{\left|\int_{-\infty }^{+\infty }s(t_{0}-\tau )\cdot h(\tau )\cdot \omega (\tau )d\tau \right|}^{2}}{N_{0}\int_{-\infty }^{+\infty }{\left|h(\tau )\cdot \omega (\tau )\right|}^{2}d\tau }$$

where ω(τ) indicates the selected window function used for weighting, the windowed SNR is subtracted from the unwindowed SNR to obtain the SNR loss as [2]:

(17) $${\mathrm{SNR}}_{\mathrm{loss}}=10\log\frac{{\mathrm{SNR}}_{\omega }}{\mathrm{SNR}}=10\log\frac{{\left|\int_{-T/2}^{T/2}w(\tau )d\tau \right|}^{2}}{T{\int_{-T/2}^{T/2}\left|w(\tau )\right|}^{2}d\tau }$$

where *T* indicates the pulse duration. For the discrete signal sampled in the time domain, the SNR loss can also be expressed as:

(18) $${\mathrm{SNR}}_{\mathrm{loss}}=10\log\frac{{\left(\sum_{n=1}^{N}w(n)\right)}^{2}}{N\sum_{n=1}^{N}{\left(w(n)\right)}^{2}}$$

where *N* indicates the number of sampling points. With the selected cosine window function, Figure 6 shows the relationship between the SNR loss and the window factor. Therefore, with the same geometric resolution, bandwidth and SLL, the SNR obtained by the proposed imaging mode can be about 3 dB higher than the conventional starring spotlight mode as shown in Figure 6, when the selected window factor *α* is 0.2.

### 3.3. Imaging Algorithms

Due to the transmitted NLFM waveform and azimuth non-uniform sampling in the proposed imaging mode, conventional SAR focusing algorithms cannot be directly applied to handle its corresponding raw data. The BPA is widely used in multiple SAR new imaging modes, especially for high resolution modes. The only change in BPA is the range frequency matched filter, which should be obtained by taking the complex conjugate of the Fourier transform of the discrete sampled NLFM waveform. The range frequency matched filter for the NLFM waveform is written as:

(19) $$H_{\mathrm{match}}(f)={\left\{\mathrm{FT}\left[s_{\mathrm{NLFM}}(\tau )\right]\right\}}^{*}$$

where $s_{\mathrm{NLFM}}(\tau )$ represents the azimuth nonlinear frequency modulation signal, $\mathrm{FT}\left[\cdot \right]$ represents the Fourier transform operator, and ${\left\{\cdot \right\}}^{*}$ indicates the conjugate operation.

**Figure 7:**
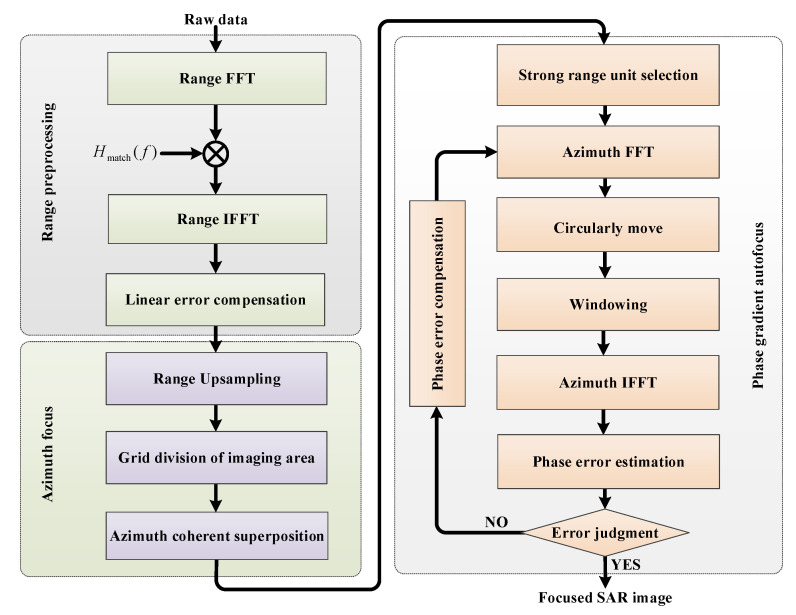
Block diagram of the modified BPA for the proposed imaging mode.

Furthermore, in airborne SAR imaging, motion compensation (MoCo) must be considered during SAR focusing. The BPA with MoCo for the proposed imaging mode is shown in Figure 7. After range pulse compression, the preliminary linear offset error is taken according to the radar platform trajectory information compensation, which is obtained through the inertial navigation system [22]. After azimuth coherent superposition for each pixel in the imaged scene, the coarse focused complex image is obtained, and the residual azimuth modulation phase error is compensated through the phase gradient autofocus (PGA) algorithm [23,24]. In the PGA algorithm, the range units with high signal-to-clutter ratio (SCR) are selected to improve the azimuth phase error estimation accuracy. After azimuth tast Fourier transform of the selected range units, the azimuth spectrum is circularly moved towards the Doppler centroid, and window filtering of the resulting data removes clutter signals and improves the SCR. After circular shifting and windowing, the linear unbiased minimum variance is used to calculate the phase error gradient after the inverse Fourier transform, and the well focused SAR images are output while the phase error is less than the designed error threshold.

**Figure 8:**
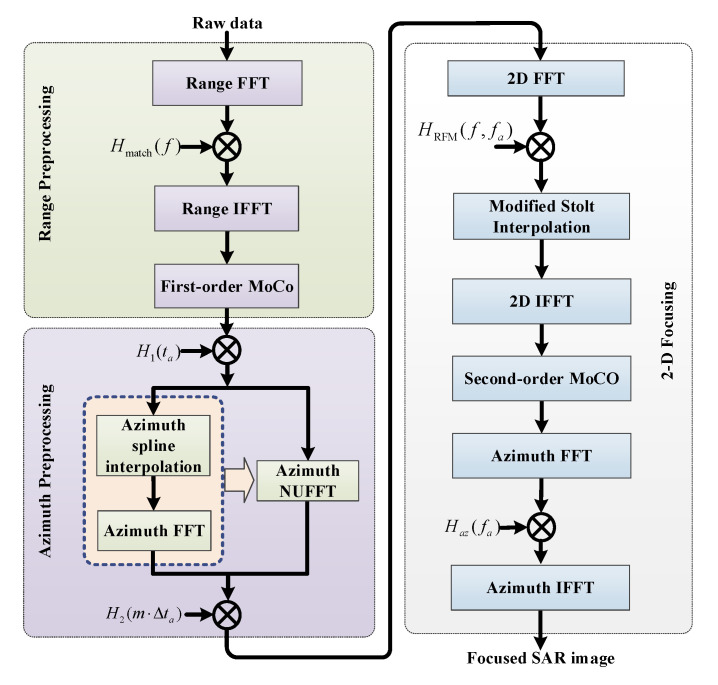
Block diagram of the modified RMA processor for the proposed mode.

The major disadvantage of the BPA is low computational efficiency, while frequency domain algorithms [25,26,27] show more computational efficiency and are widely adopted in recent SAR imaging modes. However, to handle the raw data of the new proposed modes, these algorithms usually need to be modified. RMA is used for images since almost no approximation is introduced in the formula derivation. The modified RMA for the proposed starring spotlight mode with NLFM and ANUS is shown in Figure 8, which includes three major steps: range preprocessing for NLFM, azimuth sampling adjustment, and 2D focusing.

As shown in Figure 8, the range preprocessing step includes NLFM pulse compression and the first-order MoCo. In airborne SAR imaging, MoCo must be considered to improve focusing performances, and the two-step MoCo method is widely adopted in multiple airborne SAR imaging modes [28,29]. After range frequency matched filtering with (19), the first-order MoCo is carried out to eliminate range-dependent linear motion errors.

In the starring spotlight mode, the azimuth beam is steered from fore to aft to extend the Doppler bandwidth and improve the azimuth resolution, and the operated PRF would be smaller than the extended Doppler bandwidth, which results in Doppler spectrum aliasing after azimuth Fourier transform. Azimuth resampling in the two-step focusing technique, which introduces an azimuth convolution between the raw data and the selected chirp signal, can resolve the Doppler aliasing problem. The azimuth convolution is implemented by two complex matrix multiplications and one Fourier transform. The first transfer function for multiplication is expressed as [30]:

(20) $$H_{1}(t_{a})=\exp\left(j2\pi \frac{v^{2}t_{a}^{2}}{\lambda R_{c}}\right)$$

where v is the moving speed of the radar, and $R_{c}$ is the slant range from the flight path to the imaged scene center. As the azimuth time $t_{a}$ is non-uniformly sampled in the proposed mode, the following azimuth Fourier transform cannot be directly applied before azimuth interpolation to obtain the azimuth uniform sampled raw data. Spline interpolation is a useful method to obtain uniform sampled raw data from non-uniform sampled data but with the low efficiency [31,32,33]. Non-uniform fast Fourier transform (NUFFT) could be instead of azimuth interpolation and FFT to improve the computational efficiency as shown in Figure 8. Consequently, the second transfer function for multiplication in azimuth pre-filtering is expressed as [34]:

(21) $$H_{2}(m\cdot \Delta t_{a})=\exp\left[j2\pi \frac{v^{2}{\left(m\cdot \Delta t_{a}\right)}^{2}}{\lambda R_{c}}\right],\mathrm{with}\;m=-P/2,\ldots ,P/2-1$$

where $\Delta t_{a}\leq 1/B_{\mathrm{total}}$ indicates the azimuth time sampling interval after upsampling in azimuth pre-filtering, $B_{\mathrm{total}}$ is the total Doppler bandwidth of the imaged scene, and P is the number of azimuth samples after upsampling. In order to efficiently apply FFT to implement azimuth convolution between the SAR raw data and the selected chirp signal, *P* should be [30]:

(22) $$P=\frac{\lambda R_{c}}{2v^{2}\cdot \Delta t\cdot \Delta t_{a}}$$

where Δt is the original average azimuth time sampling interval.

After azimuth resampling to overcome problems of Doppler spectrum aliasing and azimuth non-uniform sampling in the proposed imaging mode, the resulting raw data could be handled by the modified RMA processor. The first step of the modified RMA is the reference function multiplication in the 2D frequency domain, and the reference function is expressed as [35]:

(23) $$H_{\mathrm{RFM}}(f,f_{a})=\exp\left(\frac{4\pi R_{\mathrm{ref}}}{c}\sqrt{{\left(f_{0}+f\right)}^{2}-\frac{c^{2}f_{a}^{2}}{4v^{2}}}\right)$$

where $R_{\mathrm{ref}}$ represents the reference distance. Afterwards, inorder to implement the range dependent motion compensation by second-order MoCo in the 2D time domain, the modified stolt interpolation is carried out, which can separate range cell migration correction (RCMC) from azimuth compression in RMA. The modified stolt interpolation can be described as [36]:

(24) $$f_{1}=\sqrt{{\left(f_{c}+f\right)}^{2}-\frac{c^{2}f_{a}^{2}}{4v^{2}}}-\sqrt{f_{c}^{2}-\frac{c^{2}f_{a}^{2}}{4v^{2}}}$$

where $f_{1}$ represents the new range frequency after interpolation. After the modified stolt interpolation, the second-order MoCo is performed in the 2D time domain. Finally, the azimuth compression is carried out in the range Doppler domain, and the azimuth matched filtering function is expressed:

(25) $$H_{az}(r,f_{a})=\exp\left\{j\frac{4\pi (r-R_{ref})}{c}\sqrt{f_{c}{}^{2}-\frac{c^{2}f_{a}^{2}}{4v^{2}}}\right\}\cdot \exp\left(j\pi \frac{\lambda R_{c}f_{a}^{2}}{2v^{2}}\right)$$

The first term is multiplied to implement azimuth compression, while the second one is taken to eliminate the azimuth modulation introduced by the processing step of azimuth sampling adjustment.

## 4. Simulation and Results Analysis

**Figure 9:**
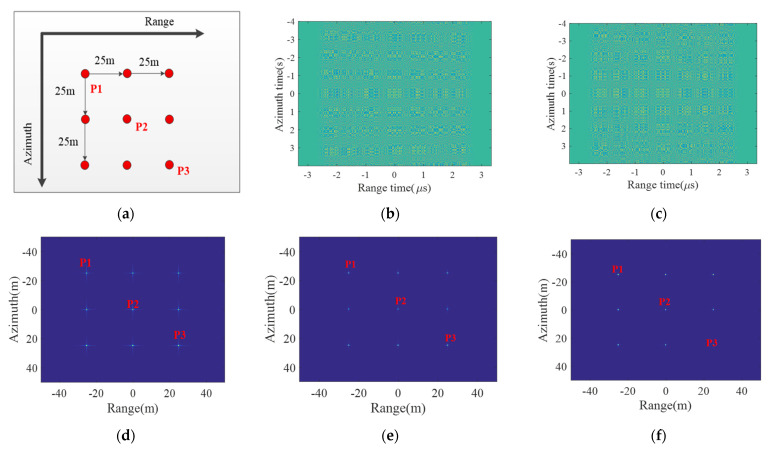
Imaging results of the designed scene with nine point targets. (**a**) The designed scene with nine point targets. (**b**) Real part of echoes in the conventional starring spotlight mode. (**c**) Real part of echoes in the proposed starring spotlight mode. (**d**) Focused image in the conventional mode. (**e**) Focused image with window weighting in the conventional mode. (**f**) Focused image in the proposed mode.

To validate the proposed high-resolution imaging mode with low SLL without window weighting, a simulation experiment on a designed scene with nine point targets is carried out, and the arrangement of nine point targets is shown in Figure 9a. Point target P2 is located at the center of the designed scene, while the distance between adjacent point targets in the both range and azimuth directions is 25 m, and simulation parameters are listed in Table 1.

Echoes of the designed scene with nine point targets in the conventional starring spotlight and the proposed mode are shown in Figure 9b,c, respectively, while the raised cosine window factor for both NLFM generation and ANUS design in the proposed imaging mode is 0.3. Using the modified RMA processor as shown in Figure 8 without window weighting to handle the raw data, imaging results of both modes are shown in Figure 9c,f. Strong sidelobes of point targets could be obviously observed in Figure 9d, while sidelobes are well suppressed after window weighting in the conventional mode as shown in Figure 9e. However, without any window weighting in the both range and azimuth dimensions, sidelobes are also well suppressed in the proposed low sidelobe imaging mode as shown in Figure 9f.

**Figure 10:**
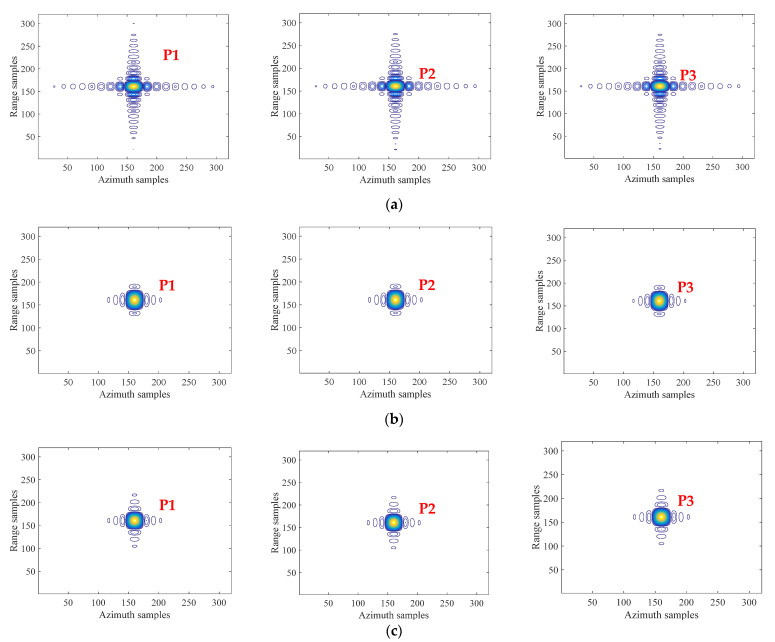
Contour plots of imaged point targets. (**a**) Without windowing in the conventional mode. (**b**) With windowing in the conventional mode. (**c**) Without windowing in the proposed mode.

Contour plots of three point targets P1, P2, and P3 in Figure 9 are shown and compared in Figure 10, and their corresponding measured focusing parameters including geometric resolution, peak sidelobe ratio (PSLR) and integral sidelobe ratio (ISLR) are summarized and listed in Table 2. Compared with imaging results in the conventional starring spotlight mode, the geometric resolution will be slightly increased in the both range and azimuth directions in the proposed imaging mode, while the SLL including PSLR and ISLR will be obviously reduced similar to window weighting. However, window weighting for such sidelobe suppression with performances listed in Table will lead to about 3 dB SNR reduction in the final focused SAR image. Furthermore, when the same window weighting function is used for NLFM waveform generation and ANUS design, the SLL is better in the range direction than in azimuth, since the Gibbs effect in the small time-bandwidth product case is more obvious, which will reduce the sidelobe suppression effect.

**Figure 11:**
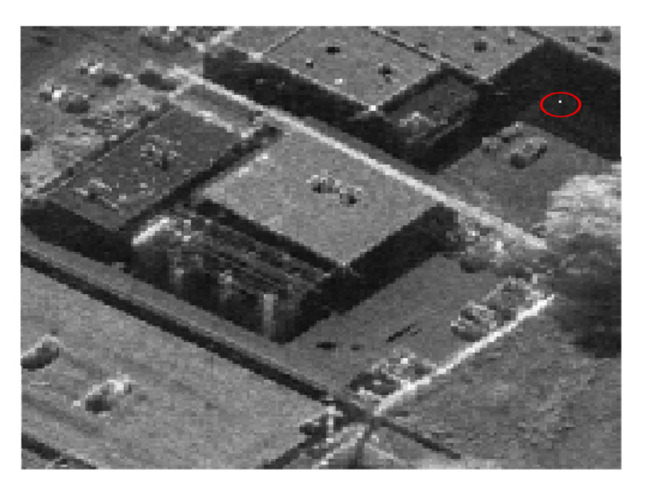
Focused SAR image with 0.3 m resolution for simulation.

**Figure 12:**
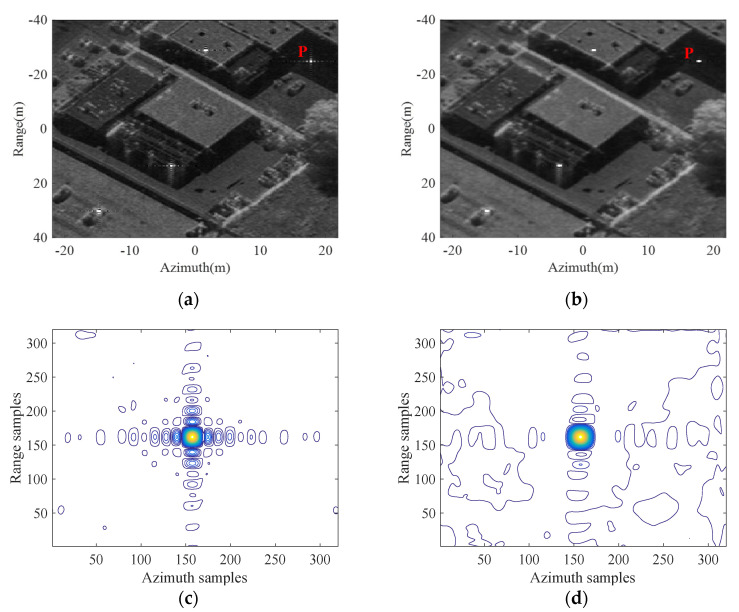
Distributed target simulation results. (**a**) The imaging result in the conventional starring spotlight mode. (**b**) The imaging result in the proposed mode. (**c**) Contour plots of P in the starring spotlight mode. (**d**) Contour plots of P in the proposed mode.

In order to further verify the superiority of the proposed imaging mode, a focused SAR image as shown in Figure 11 with 0.3 m resolution is selected as the reflectivity map for echo simulation and imaging process. Imaging simulation results of the designed scene in the conventional and proposed starring spotlight modes are shown in Figure 12a,b, while sidelobes in the proposed mode as shown in Figure 12a are obviously suppressed compared with the focused SAR image in the conventional starring spotlight mode as shown in Figure 12b, especially for strong scattering points. The strong scattering point P in both modes as shown in Figure 12a,b are interpolated, while their corresponding contour plots are shown in Figure 12c,d.

Measured focusing parameters of the point target P are computed and listed in Table 3. The SLL in range is originally about −14 dB and reduced to about −22 dB due to the NFLM signal as shown in Table 3. Furthermore, because of azimuth non-uniform sampling, the azimuth SLL of the point target P drops from −14 dB to about −20 dB.

**Figure 13:**
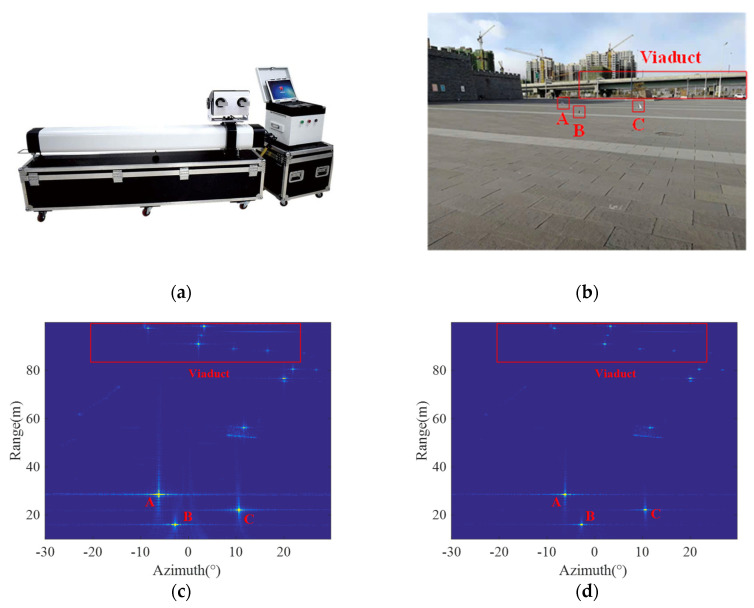

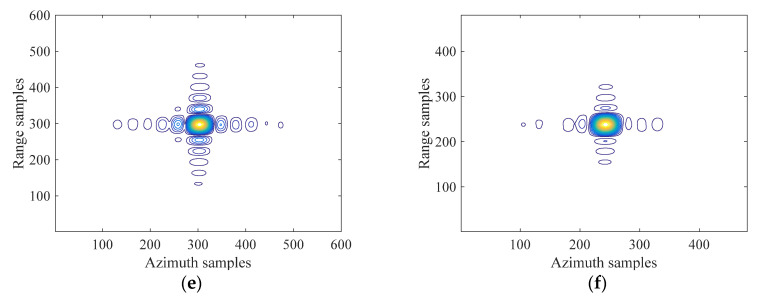
Experiments via a ground−based SAR system. (**a**) The ground−based SAR system for experiments. (**b**) Experimental site with three corner reflectors. (**c**) Imaging result of the conventional mode. (**d**) Imaging result of the proposed mode. (**e**) Contour plots of reflector B in the conventional mode. (**f**) Contour plots of reflector B in the proposed mode.

In addition, a ground-based SAR system was used to conduct experiments on the conventional imaging mode with LFM and uniform sampling and the proposed imaging modes with NLFM and ANUS. Figure 13a shows the ground-based SAR system used for experiments, which is named as MPDMR-02-LSA1707 ground-based micro-deformation monitoring radar produced by Inner Mongolia Mypattern Technology Co., Ltd. in Inner Mongolia, China [37]. Three corner reflectors are arranged as targets in the open field in the front of the radar as shown in Figure 13b. Imaging results of the conventional mode and the proposed are shown in Figure 13c,d, respectively, and it can be seen that sidelobes are generally reduced in Figure 13d, and the location of the point target is more specific. Finally, the resolution, PSLR, and ISLR of the diagonal reflector B are measured and summarized in Table 4. The imaging results on the experimental real raw data are consistent with the simulation results in Figure 10 and Figure 12. According to measured parameters listed in Table 2, Table 3 and Table 4, the SLL in the proposed mode is obviously suppressed with a slightly broadened resolution similar to window weighting, but the SNR reduction due to window weighting is avoided.

## 5. Conclusions

The high sidelobe level will degrade the SAR image quality, and window weighting is usually adopted during focusing process, which will introduce two problems: resolution slightly broadening and SNR reduction. The novel starring spotlight mode with NLFM and ANUS is proposed for low sidelobe imaging. The NLFM signal is transmitted instead of the conventional LFM signal for low sidelobes in range, while the ANUS behavior (dense in middle and sparse in both sides) is adopted for low sidelobes in azimuth. Compared with the conventional starring spotlight mode with 2D window weighting for the same low sidelobes, the geometric resolution in the proposed mode is also slightly broadened, but the SNR reduction in the final focused SAR images due to 2D window weighting is avoided. For the same low side-lobes and resolution, the SNR of the obtained SAR image in the proposed mode is about 3 dB higher than 2D window weighting. In other words, for the same image quality, the proposed imaging mode could save about one-half of the transmitting power.

## Figures and Tables

**Table 1:** Simulation parameters.

Parameter	Value
Pulse duration	5 μs
Pulse bandwidth	500 MHz
Sampling frequency	600 MHz
Carrier frequency	9.6 GHz
Sensor velocity	150 m/s
Length of antenna	2 m
Slant range to the scene center	30 km
Azimuth illumination time	8 s
Number of azimuth samples	8000
Azimuth average sampling interval	1 ms

**Table 2:** Measured focusing parameter of imaging results on the designed scene.

Imaging Mode	Target	Range			Azimuth		
		Resolution (m)	PSLR (dB)	ISLR (dB)	Resolution (m)	PSLR (dB)	ISLR (dB)
The conventional mode	P1	0.346	−13.242	−10.106	0.264	−13.275	−10.243
	P2	0.346	−13.244	−10.106	0.264	−13.276	−10.244
	P3	0.346	−13.243	−10.106	0.264	−13.276	−10.244
Two-dimensional windowing	P1	0.450	−22.959	−20.489	0.312	−20.292	−18.626
	P2	0.450	−22.964	−20.871	0.312	−20.319	−18.489
	P3	0.450	−22.891	−20.814	0.312	−20.891	−18.644
The proposed mode	P1	0.406	−20.2314	−18.519	0.312	−20.262	−18.538
	P2	0.404	−20.2304	−18.517	0.312	−20.118	−18.544
	P3	0.404	−20.228	−18.516	0.312	−20.126	−18.516

**Table 3:** Measured focusing parameters of the point target P in Figure 11.

Imaging Mode	Range			Azimuth		
	Resolution (m)	PSLR (dB)	ISLR (dB)	Resolution (m)	PSLR (dB)	ISLR (dB)
The conventional mode	0.350	−14.462	−10.122	0.272	−13.960	−10.122
The proposed mode	0.414	−22.046	−14.532	0.336	−19.649	−14.390

**Table 4:** Measured focusing parameters of diagonal reflector B in Figure 13.

Imaging Mode	Range			Azimuth		
	Resolution (m)	PSLR (dB)	ISLR (dB)	Resolution (°)	PSLR (dB)	ISLR (dB)
The conventional mode	0.262	−12.631	−10.134	0.280	−12.080	−10.115
The proposed mode	0.294	−19.148	−16.354	0.313	−17.881	−15.354

## Data Availability

Not applicable.
